# WEB-BASED PHYSICAL ACTIVITY INTERVENTION IN MIDDLE-AGED AND OLDER ADULT VETERANS WITH COPD

**DOI:** 10.1093/geroni/igz038.1608

**Published:** 2019-11-08

**Authors:** Stephanie A Robinson, Stephanie A Robinson, Deanna L Mori, Caroline R Richardson, Marilyn L Moy

**Affiliations:** 1 Edith Nourse Rogers Memorial Veterans Hospital, Bedford, Massachusetts, United States; 2 VA Boston Healthcare System, Boston, Massachusetts, United States; 3 University of Michigan, Ann Arbor, Michigan, United States; 4 VA Boston Healthcare System, West Roxbury, Massachusetts, United States

## Abstract

Physical activity (PA) is recommended in all patients with chronic obstructive pulmonary disease (COPD). Technology-based interventions can deliver effective, scalable behavior-change techniques; though feasibility and acceptability among older adults is not established. Veterans with COPD (N=112, aged 49-89 years, median=68) were randomized to a 12-week web-based and pedometer intervention or a pedometer alone (control). Across groups, there was no significant difference between middle-aged (<68 years) and older (≥68 years) adults in percentage of pedometer wear-days over the study period (83.6% vs. 89.9%). In the intervention, there were no significant differences between middle-aged and older adults in total number of website logons (15.04 vs. 16.31), or proportion who reported they recommended the intervention (96.4% vs. 96.7%), found it easy to use (93.1% vs. 90.0%), and would continue to walk (93.1% vs. 89.7%). We conclude that a web-based PA intervention with a pedometer is feasible and acceptable in an older COPD population.

